# Computer-Aided Design of *Plasmodium chabaudi*-Derived Peptides with Dual Antibiofilm and Anti-inflammatory Activities

**DOI:** 10.1021/acsmedchemlett.6c00007

**Published:** 2026-05-05

**Authors:** Raquel M. Quigua Orozco, Alexandre D. O. Santos, Joelma P. Rossetto, Elisângela S. Madalozzo, Lívia V. Luchi, Samilla B. Rezende, Lai Yue Chan, Danieli F. Buccini, Maria L. R. Macedo, Angela Mehta, David J. Craik, Octávio L. Franco, Marlon H. Cardoso

**Affiliations:** ∇ S-Inova Biotech, Programa de Pós-Graduação em Biotecnologia, 186072 Universidade Católica Dom Bosco, Campo Grande, Mato Grosso do Sul 79117-900, Brazil; ‡ 54534 Universidade Federal de Mato Grosso do Sul (UFMS), Campo Grande, Mato Grosso do Sul 79070-900, Brazil; § Universidade Estadual de Mato Grosso do Sul (UEMS), Naviraí, Bairro Santo Amaro CEP 79115-898, Brazil; ∥ Institute for Molecular Bioscience, Australian Research Council Centre of Excellence for Innovations in Peptide and Protein Science, 1974 The University of Queensland, Brisbane 4072, Australia; ⊥ Laboratório de Purificação de Proteínas e suas Funções Biológicas, Universidade Federal de Mato Grosso do Sul, Campo Grande, Mato Grosso do Sul 79070-900, Brazil; # Embrapa Recursos Genéticos e Biotecnologia, Brasília 70.770-917, Brazil; 7 Centro de Análises Proteômicas e Bioquímicas, Programa de Pós-graduação em Ciências Genômicas e Biotecnologia, Universidade Católica de Brasília, Brasília 71966-700, Brazil; 8 Programa de Pós-Graduação em Ciências Ambientais e Sustentabilidade Agropecuária, 186072 Universidade Católica Dom Bosco, Campo Grande, Mato Grosso do Sul 79117-900, Brazil

**Keywords:** Antimicrobial peptides, biofilm inhibition, immunomodulatory activity, drug design

## Abstract

Advances in computational design have greatly accelerated antimicrobial peptide engineering. In this study, three *Plasmodium chabaudi*-derived peptides (PcDBS1R1, PcDBS1R5, and PcDBS1R9), generated using the Joker computational design algorithm, were synthesized and characterized for their structural and functional properties. Biophysical analyses revealed that PcDBS1R5 and PcDBS1R9 predominantly adopted α-helical structures with high amphipathicity, whereas PcDBS1R1 exhibited greater structural plasticity. PcDBS1R5 and PcDBS1R9 displayed antibacterial activity against an *Acinetobacter baumannii* clinical isolate, whereas PcDBS1R1 showed pronounced antibiofilm effects. None of the peptides exhibited cytotoxicity toward murine macrophages, and all significantly reduced nitric oxide production in lipopolysaccharide-stimulated macrophages, suggesting potential anti-inflammatory activity. Overall, these findings demonstrate that computer-aided design of *P. chabaudi*-derived peptides can yield molecules with antibiofilm, and immunomodulatory properties, minimal cytotoxicity, and promising therapeutic potential as scaffolds for next-generation peptide-based treatments targeting biofilm-associated bacterial infections.

## Graphical abstract

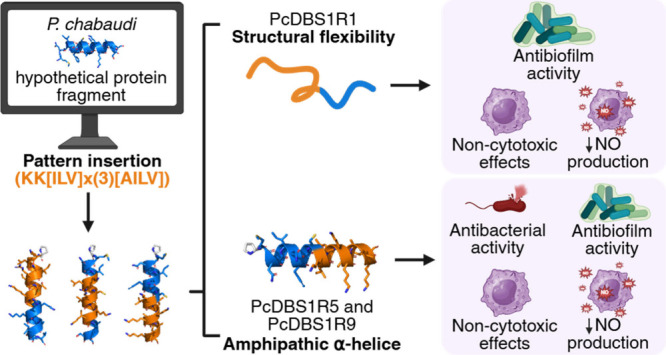

Bacterial infections are a notorious threat across the world, exerting a profound impact on human life.^1^ They are caused by pathogenic bacteria in their free-swimming planktonic form or through the formation of complex multicellular aggregates known as biofilms.^2^ Biofilms are organized microbial communities, commonly composed of bacteria embedded within a self-produced matrix of extracellular polymeric substances consisting of polysaccharides, proteins, lipids, and extracellular DNA.^3,4^

Bacterial biofilms are directly related to serious clinical conditions and difficult-to-treat infections, posing a significant challenge in the era of antimicrobial resistance.^5^ According to estimates from the National Institutes of Health and the Centers for Disease Control and Prevention, biofilms are implicated in approximately 65–80% of all human infections.^3,4,6^ This alarming prevalence, combined with the scarcity of effective therapeutic strategies against biofilm-associated infections, underscores the urgent need for novel agents with potent inhibitory effects on biofilm-forming bacteria.^6,7^

As a promising alternative to conventional antibiotics, antimicrobial peptides (AMPs) have attracted substantial attention worldwide.^8^ They hold great potential as therapeutic molecules due to their potent and broad-spectrum activity, structural diversity, and distinct modes of action against pathogenic microorganisms.^9^ Owing to these structural and biological properties, particularly their strong antimicrobial potential, AMPs have been widely employed as input molecules for computational strategies aimed at designing synthetic and optimized peptide analogues.^10^ Such strategies seek to enhance antimicrobial efficacy, improve stability and bioavailability, minimize cytotoxicity, and reduce production costs.^10^

Recent advances in bioinformatics and computational biology have greatly accelerated peptide discovery and optimization. Tools based on genetic algorithms, pattern recognition, and *de novo* design have been used to generate novel AMPs with improved potency.^11^ Moreover, the integration of machine learning and deep learning techniques has revolutionized peptide discovery, allowing for accurate prediction of diverse qualities (e.g., antimicrobial activity, toxicity, resistance development, physicochemical properties, or structure).^12,13^ These data-driven approaches not only accelerate AMP design but also facilitate the identification of novel sequences with enhanced therapeutic potential against multidrug-resistant pathogens.^12,14^

Among the bioinformatics methodologies available for generating optimized AMPs, the Joker algorithm stands out for its ability to design functional peptides by inserting antimicrobial sequence patterns into AMP and non-AMP scaffolds.^15^ During its development, the α-helical pattern (KK­[ILV]­x(3)­[AILV]) was obtained from 248 α-helical AMPs deposited in the Antimicrobial Peptide Database. Moreover, the blind set to be used as input for the Joker algorithm was composed of sequences with no information about their antimicrobial activities,^15^ including a hypothetical protein fragment (GenBank: CAH75736.1 = MNAINFTCTVHKKVAISV) from *Plasmodium chabaudi*. This non-AMP sequence was selected for the current study due to its propensity to adopt at least three stable turns of α-helix, and for not being characterized previously. The Joker algorithm was applied by inserting the antimicrobial pattern (KK­[ILV]­x(3)­[AILV]) into the input sequence (GenBank: CAH75736.1 = MNAINFTCTVHKKVAISV) in a sliding-window fashion, resulting in nine variants, named PcDBS1R1 to PcDBS1R9 (**Pc**: *P. chabaudi*; **DBS**: database sequence 1; **R1 to R9**: generation). In a preliminary study,^15^ all the variants were tested against a bioluminescent *Pseudomonas aeruginosa* strain and against human erythrocytes (Table 1). Among the variants, PcDBS1R1, PcDBS1R5 and PcDBS1R9 were the most effective in terms of high antibacterial activity, measured as minimal inhibitory concentration (MIC), and minimal hemolytic effects at the highest concentration tested (Table 1; Figure 1). Molecular modeling studies revealed that all variants have four turns of α-helix in their structure, but have different hydrophobic moments (<μH>) and, therefore, varying levels of amphipathicity. Structural statistics are given in Table S1, including stereochemical and fold quality for the theoretical models here generated using AlphaFold2.^16^

**Table 1:** Antibacterial Activity against a Bioluminescent *P. aeruginosa* H1001, Hemolytic Activity and Physicochemical Properties of PcDBS1-Derived Peptides[Table-fn t1fn6]

Peptide	Sequence	MIC^a^ [Table-fn t1fn7]	HC_50_ ^b^ [Table-fn t1fn7]	z^c^	H^d^	μH^e^
PcDBS1R1	P**KLAIRITCKI**HKKVAISV	6	>92	+5	50.5	0.293
PcDBS1R2	PM**KLLNRICTKI**KKVAISV	11.5	>92	+5	51.0	0.551
PcDBS1R3	PMN**KILFRITVKI**KVAISV	92	>92	+4	64.5	0.318
PcDBS1R4	PMNA**KILTRIVHKI**VAISV	47	>92	+3	62.6	0.274
PcDBS1R5	PMNAI**KLLCRVHKKI**AISV	6	47	+4	57.2	0.424
PcDBS1R6	PMNAIN**KILTRIKKKI**ISV	11.5	>92	+5	43.3	0.588
PcDBS1R7	PMNAINF**KILVRIKVKI**SV	23	>92	+4	59.9	0.126
PcDBS1R8	PMNAINFT**KILHKIVAKI**V	23	>92	+3	62.7	0.397
PcDBS1R9	PMNAINFTC**KLLKKVAIKI**	6	>92	+4	58.0	0.439

a MIC: minimal inhibitory concentration (μmol L^–1^).

b HC_50_: concentration required to cause 50% hemolysis of red blood cells (μmol L^–1^).

c Charge.

d Mean hydrophobicity.

e Mean hydrophobic moment. The hydrophobic moment was calculated according to the Eisenberg scale.

† The physicochemical properties were calculated on the *HeliQuest* server. The α-helical antimicrobial amino acid pattern is shown in bold type.

* Data published by Porto and co-workers.^15^

**Figure 1:**
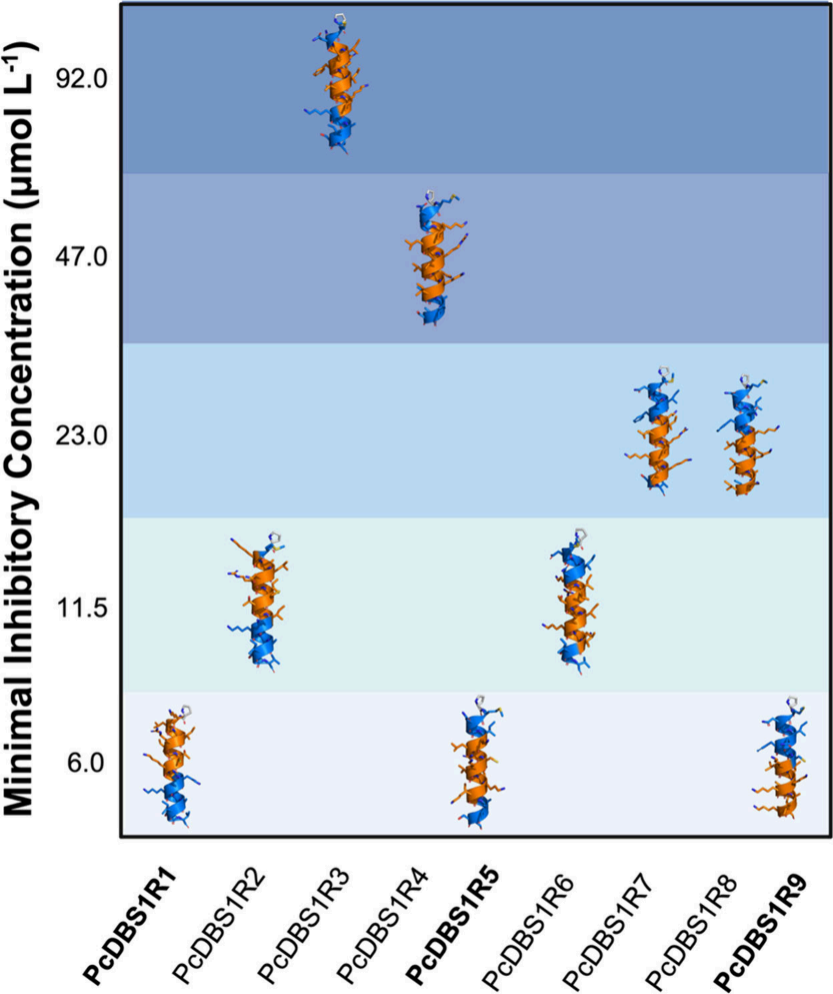
Molecular modeling of *P. chabaudi* protein fragment variants generated by the Joker algorithm and their respective MICs against a bioluminescent *P. aeruginosa* H1001 strain. The orange segments represent where the α-helical pattern KK­[ILV]­x(3)­[AILV] was inserted. Blue regions resemble the original ribosomal protein fragment from *P. chabaudi* (MNAINFTCTVHKKVAISV). Proline (colored white) was used for N-terminal capping in all variants. PcDBS1R1, PcDBSR5 and PcDBS1R9 had the lowest MIC values and were selected for further studies.

Building upon these findings, the selected peptides, including PcDBS1R1, PcDBS1R5 and PcDBS1R9, were chemically synthesized and purchased from Peptide 2.0 Incorporated (Chantilly, VA, USA) to investigate their three-dimensional structures and biological activities, with a core focus on bacterial biofilms and anti-inflammatory effects. Structures were characterized by circular dichroism (CD) and nuclear magnetic resonance (NMR), and biological activities were determined by inhibitory activity assays against planktonic cells and biofilms of the American Type Culture Collection (ATCC) and antibiotic-resistant *Acinetobacter baumannii* and *Klebsiella pneumoniae* strains. The peptides were also evaluated for their cytotoxic potential against murine macrophages, as well as their potential in modulating macrophages nitric oxide (NO) production.

Molecular masses were verified by matrix-assisted laser desorption/ionization time-of-flight mass spectrometry (MALDI-TOF MS), with masses of 2120.44, 2135.17, and 2147.26 Da for PcDBS1R1, PcDBS1R5 and PcDBS1R9, respectively (Figures S1, S2 and S3). Physicochemical analyses were performed using the *HeliQuest* server, indicating high net positive charges ranging from +3 to +5, moderate hydrophobicity, and high hydrophobic moments, particularly for PcDBS1R5 and PcDBS1R9 (Table 1).

CD analysis was conducted to study the secondary structure of PcDBS1R1, PcDBS1R5, and PcDBS1R9 peptides in four mimetic environments, including ultrapure water, potassium phosphate buffer (10 mmol L^–1^, pH 7.2), sodium dodecyl sulfate (SDS) micelles (50 mmol L^–1^), and 2,2,2 trifluoroethanol (TFE) (30% in water (v/v)). As depicted in Figures 2A–C, all three peptides displayed random coil structures in water and buffer solutions. By contrast, in the presence of 30% TFE and SDS micelles, the peptides adopted α-helix structures (Table 2). An exception was observed for PcDBS1R1 in SDS micelles, which showed a CD signature characteristic of both α-helix and β-structures, suggesting structural plasticity compared to PcDBS1R5 and PcDBS1R9 (Figure 2A). Helicity percentages were estimated from the ellipticity value at 222 nm^17^ (Table 2).

**Figure 2:**
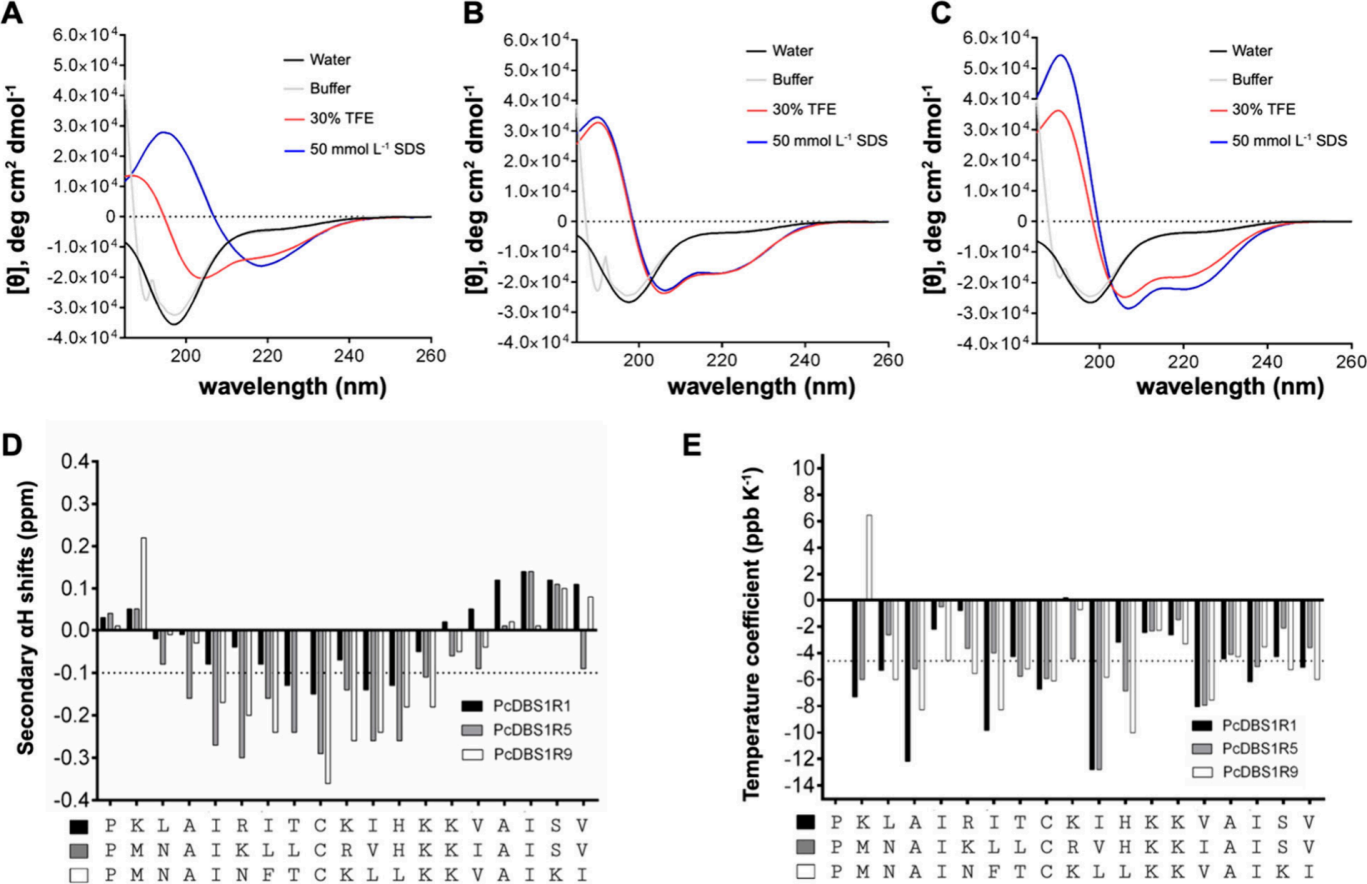
CD and NMR analyses. CD spectra of PcDBS1R1 (A), PcDBS1R5 (B), and PcDBS1R9 (C) were recorded in ultrapure water (black), 10 mmol L^–1^ potassium phosphate buffer (gray), 30% TFE (red), and 50 mmol L^–1^ SDS micelles (blue). NMR Secondary αH chemical shifts (D) and temperature coefficients (E) were measured in 30% TFE. Chemical shift deviations more negative than −0.1 ppm (dashed line in D) indicate α-helical formation, whereas amide temperature coefficient values more positive than −4.6 ppb K^–1^ (dashed line in E) suggest intrapeptide hydrogen bonding.

**Table 2:** Helicity Percentages for PcDBS1R1, PcDBS1R5 and PcDBS1R9 in Hydrophilic, Hydrophobic, and Membrane Mimetic Conditions^a^

**Solvent**	**PcDBS1R1 (%)**	**PcDBS1R5 (%)**	**PcDBS1R9 (%)**
Ultrapure water	12.48	10.96	10.94
10 mmol L^–1^ KH_2_PO_4_	13.00	10.31	10.35
30% TFE	36.54	49.15	52.03
50 mmol L^–1^ SDS	44.24	48.86	64.64

a Helicity percentages were calculated from the mean residue ellipticity at 222 nm ([θ]­222).^17^

The ability of peptides to undergo coil-to-helix transitions is known to be highly dependent on solvent polarity and environmental conditions.^18^ Consequently, peptides may adopt distinct structures depending on the physicochemical surroundings.^19^ CD spectroscopy reflects the predominant conformation of peptides in solution, whereas AlphaFold2 predicts the most thermodynamically stable conformation, typically in a vacuum or under ideal conditions. Therefore, the divergence between the experimentally observed α/β-structure and the computationally predicted α-helix likely reflects the intrinsic flexibility of PcDBS1R1, enabling structural adaptation to different environments and intermolecular interactions. This suggestion is further supported by our temperature coefficient data (Figure 2E), which identified PcDBS1R1 as the most flexible of the three peptides tested, as described below.

NMR analysis was used to further characterize the structures of the peptides. Secondary αH chemical shifts revealed that the peptides have a propensity to adopt secondary structures, including α-helices of different lengths (Figure 2D). NMR spectra were acquired at a peptide concentration of 1 mmol L^–1^ in a solvent mixture of 60% H_2_O, 30% TFE-*d*_3_, and 10% D_2_O (v/v) at pH 4.3, as described by Cardoso and co-workers.^20^ TFE is commonly used for studying the secondary structure of peptides as it displaces water molecules around the peptide backbone, favoring folding. Intrapeptide hydrogen bonds were identified through temperature coefficient analysis of the amide proton chemical shifts (Figure 2E). PcDBS1R1 had only a short α-helical segment from Thr8 and His12, confirming its structural plasticity. By contrast, the remaining peptides adopted extended and well-defined α-helix regions, from Ala4 to His12 for PcDBS1R5 and from Ile5 to Lys13 for PcDBS1R9. Among the three peptides, PcDBS1R1 had the lowest α-helical content as assessed by NMR, consistent with the CD data. Chemical shift assignments, including backbone and side-chain resonances are summarized in Tables S2–S4. Temperature coefficient analyses identified residues protected from solvent exposure and, therefore, indicate amino acid residues that may be involved in intrapeptide hydrogen bonding (values more positive than – 4.6 ppb K^–1^).^21^ The temperature coefficient data also provide insights into the relative flexibility of the peptides, following the order PcDBS1R1 > PcDBS1R9 > PcDBS1R5. In summary, PcDBS1R1 is the most flexible peptide with the greatest structural plasticity, whereas PcDBS1R5 has the most rigid structural scaffold since it presents a greater number of amino acid residues protected from the solvent. Finally, PcDBS1R9 seems to represent an intermediate secondary structure in terms of flexibility/rigidity in comparison to PcDBS1R1 and PcDBS1R5. These structural features directly influence the biological activities of these peptide variants, as described below.

Bacteriostatic and bactericidal assays for the PcDBS1 peptides were performed against two *A. baumannii* strains and two *K. pneumoniae* strains considering their clinical relevance (Figure 3). Among these strains, two are clinical isolates (*A. baumannii* (HRAN 003321216) and *K. pneumoniae* (LACEN 4455550)) and antibiogram assays were performed with 11 clinically used antibiotics, including penicillin, amoxicillin, gentamicin, ampicillin, chloramphenicol, kanamycin, cefepime, imipenem, cefaclor, Meropenem and ciprofloxacin at concentrations ranging from 2 to 64 μmol L^–1^ (Figure S4). The assays revealed a high degree of resistance to all the tested antibiotics (Figure S4). Once the resistance profile of the clinical isolates was determined, bacteriostatic and bactericidal assays were performed with the peptide candidates. All the peptides exhibited bacteriostatic activity at 64 μmol L^–1^ against *A. baumannii* (HRAN 003321216) (Figure 3B). PcDBS1R5 and PcDBS1R9 displayed the highest inhibitory effects, reducing planktonic cell growth by approximately 85% and 89%, respectively, whereas PcDBS1R1 showed the lowest activity, with an inhibition of about 44%. Interestingly, no bacteriostatic or bactericidal activity was detected against *A. baumannii* ATCC 19906 or *K. pneumoniae* strains (ATCC 10031 and LACEN 4455550) at the highest peptide concentration tested (64 μmol L^–1^) (Figure 3).

**Figure 3:**
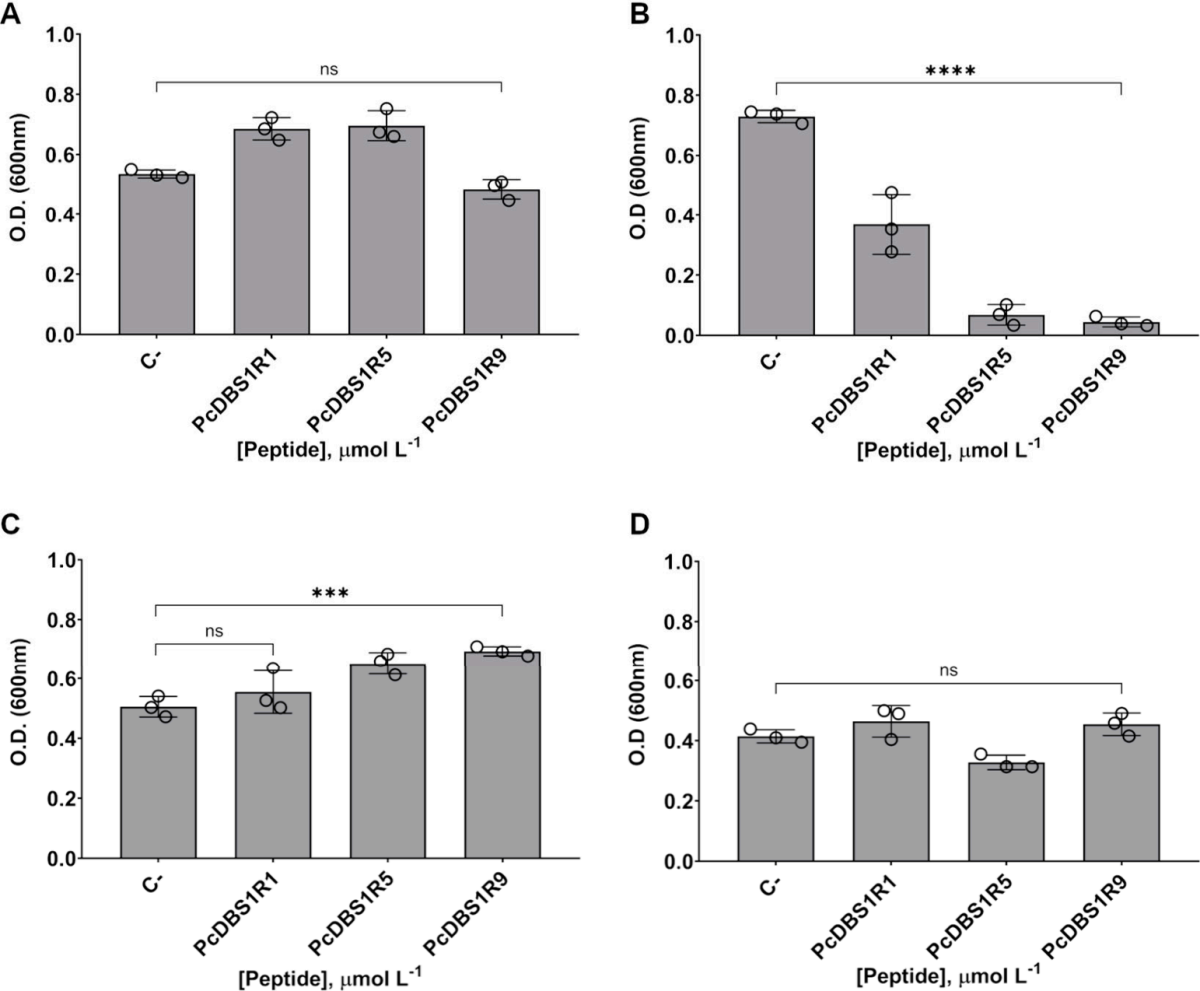
Antibacterial activities of PcDBS1R1, PcDBS1R5, and PcDBS1R9 against ATCC and antibiotic-resistant Gram-negative strains. Bacteriostatic activity assays against *A. baumannii* (ATCC 19906) (A); *A. baumannii* clinical isolate (HRAN 003321216) (B); *K. pneumoniae* (ATCC 10031) (C); and *K. pneumoniae* clinical isolate (LACEN 4455550) (D). Bar graphs represent the results at the highest peptide concentration tested (64 μmol L^–1^). C-: negative control (bacterial growth). Statistical significance was assessed using one-way analysis of variance (ANOVA), followed by Bonferroni’s multiple comparison tests (***p = 0.0004, ****p < 0.0001). Error bars represent standard deviations. ns: not significant.

The bioactivities of antimicrobial agents, including AMPs, are highly influenced by their conformational constraints and molecular plasticity.^18,22,23^ Within this context, in numerous studies the structural flexibility has been correlated with the improvement and multifunctionality of AMPs.^20,23,24^ This feature also presents an important association with amphipathicity on the influence of the peptide’s bioactivities.^23^ Here, PcDBS1R5 and PcDBS1R9 exhibited α-helical conformations, with a higher amphipathicity than PcDBS1R1 (Table 1). Moreover, both peptides have similar physicochemical properties and structural folding, although they differ in molecular flexibility. These structural features may contribute to their comparatively higher antimicrobial potency against the *A. baumannii* clinical isolate. These observations are consistent with previous studies suggesting that α-helical content, amphipathicity, and structural scaffold dynamics can influence AMP activity both *in vitro* and *in vivo*, and that distinct bacterial clinical isolates may respond differently to AMP treatment.^6,20,23,25,26^

In the present study, the inhibition of bacterial biofilm formation was evaluated in the presence of PcDBS1R1, PcDBS1R5, and PcDBS1R9 peptides. Among the tested strains, only the clinical isolates of *A. baumannii* (HRAN 003321216) and *K. pneumoniae* (LACEN 4455550) formed consistent biofilms, enabling subsequent biological investigations with these bacterial consortia. As shown in Table 3 and Figure 4, PcDBS1R5 and PcDBS1R9, at 32 μmol L^–1^, inhibited *A. baumannii* (HRAN 003321216) biofilm formation by approximately 89% and 94%, respectively (Figures 4B–C). By contrast, PcDBS1R1 inhibited biofilm formation of the same strain by about 93% at a lower concentration (16 μmol L^–1^) (Figure 4A). When tested against *K. pneumoniae* (LACEN 4455550), PcDBS1R1 exhibited an inhibition of approximately 76% at 16 μmol L^–1^ (Figure 4D). Under the same concentrations, PcDBS1R5 and PcDBS1R9 had weaker inhibitory activities of approximately 54% and 61%, respectively (Figures 4E–F). As shown in Table 3, at the highest peptide concentration tested (64 μmol L^–1^), all three peptides displayed similar levels of biofilm inhibition, ranging from 92% to 95% against *A. baumannii* (HRAN 003321216) and from 64% to 91% against *K. pneumoniae* (LACEN 4455550), thus demonstrating the high potential of these molecules as antibiofilm agents.

**Figure 4:**
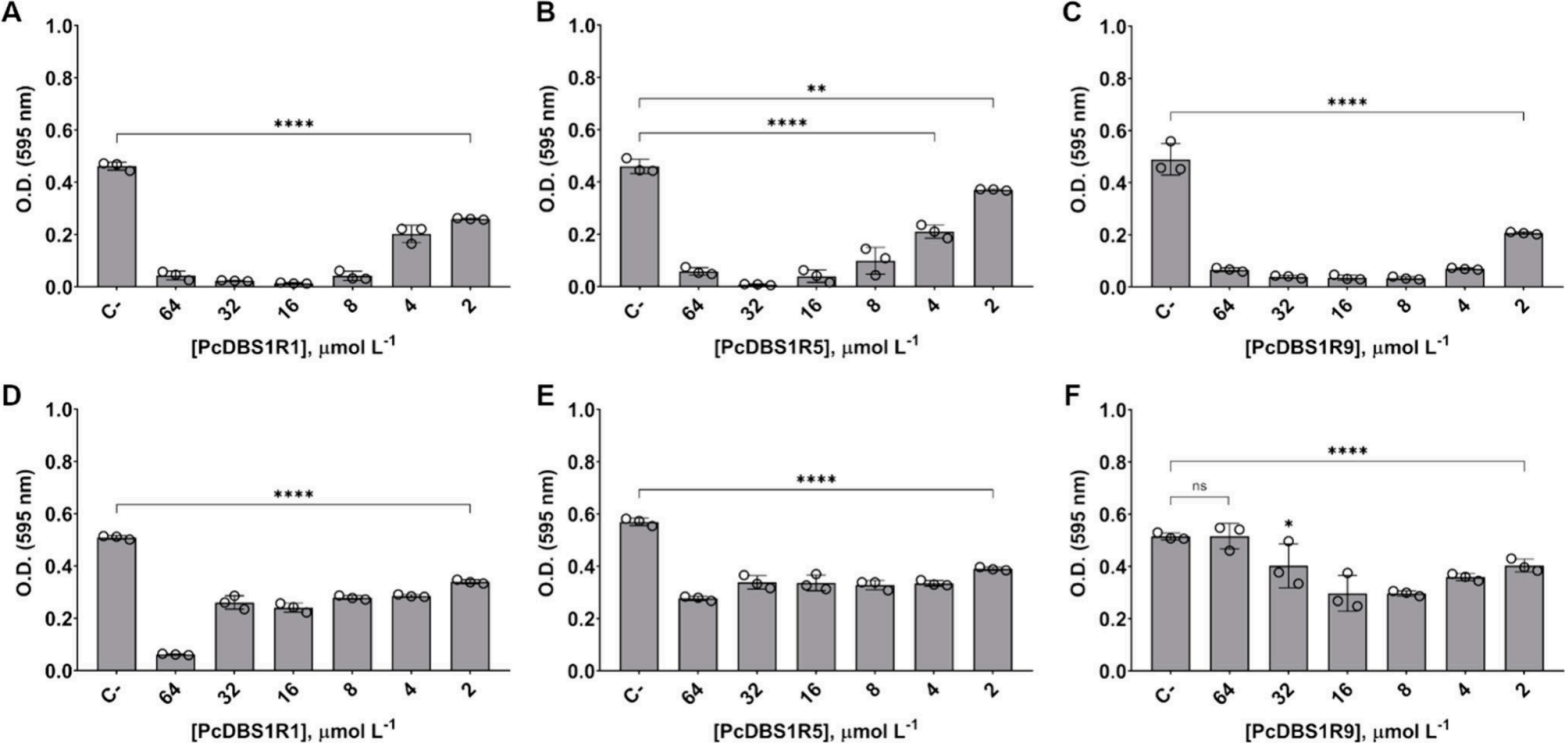
Antibiofilm activities for PcDBS1R1, PcDBS1R5, and PcDBS1R9. Biofilm inhibition assays against *A. baumannii* clinical isolate (HRAN 003321216) (A–C), and *K. pneumoniae* clinical isolate (LACEN 4455550) (D–F). C-: negative control (biofilm growth). Statistical significance was assessed using one-way analysis of variance (ANOVA), followed by Bonferroni’s multiple comparison tests (*p = 0.02, **p = 0.002, ****p < 0.0001). The error bars represent standard deviation values. ns: not significant.

**Table 3:** Antibiofilm Activity of PcDBS1R1, PcDBS1R5, and PcDBS1R9 against *A. baumannii* and *K. pneumoniae* Clinical Isolates

**Bacteria strain**	**Peptide**	**Biofilm formation inhibition** **(μmol L** ^**‑1**^ **)** ^a^	**Biofilm inhibition (%)** ^b^
*A. baumannii* **HRAN 003321216**	PcDBS1R1	16	95 ± 0.28
	PcDBS1R5	32	92 ± 0.28
	PcDBS1R9	32	94 ± 0.59
*K. pneumoniae* **LACEN 4455550**	PcDBS1R1	16	91 ± 0.28
	PcDBS1R5	32	64 ± 1.92
	PcDBS1R9	32	66 ± 2.65

a Lowest peptide concentration for maximal biofilm formation inhibition.

b Biofilm formation inhibition percentages at a peptide concentration of 64 μmol L^–1^.

Bacteria exhibit substantial differences in metabolism and resistance profiles between their planktonic and biofilm states, which significantly influences how antibacterial and antibiofilm agents, including AMPs, act under each condition.^23^ Interestingly, PcDBS1R1 showed the strongest biofilm inhibitory activity at lower concentrations against both *A. baumannii* and *K. pneumoniae*, despite its relatively weak antibacterial effect. This disconnect between antibiofilm and bacteriostatic activity has been reported for other AMPs, including LL-37 ^27^ and human β-defensin-2,^28^ supporting the notion that antibiofilm and antibacterial mechanisms may be independent.^3^ PcDBS1R1’s higher flexibility may facilitate interactions with biofilm matrix components or quorum-sensing molecules rather than directly targeting bacterial membranes, which could explain its selective antibiofilm activity.

Variability among bacterial strains, including differences in membrane composition, surface charge, and extracellular matrix structure, may further modulate peptide efficacy.^26^ Gram-negative bacteria such as *A. baumannii* and *K. pneumoniae* are known for their propensity to develop resistance to antimicrobial agents.^26,29,30^ Both species are classified by the World Health Organization as priority pathogens requiring urgent development of new therapeutic strategies.^31,32^ Our results demonstrate that the PcDBS1 derivatives evaluated here present substantial antibiofilm potential against antibiotic-resistant strains of these two bacteria, highlighting their potential as promising leads for the development of novel therapeutic agents or as scaffolds for the rational design of improved analogues.

Amphipathic α-helical conformation is widely reported as a key structural determinant for peptide’s membrane permeabilization and disruption capacity. Then, based on our results, we can hypothesize that PcDBS1R5 and PcDBS1R9 display stronger antibacterial activity against planktonic *A. baumannii*, likely due to their higher α-helical content and hydrophobic moments (amphipathicity). By contrast, the peptide PcDBS1R1, characterized by lower helical content and greater structural flexibility, showed comparatively weaker bacteriostatic activity but higher antibiofilm efficacy at lower concentrations. This observation suggests that increased conformational plasticity may facilitate interactions with biofilm matrix components (e.g., polysaccharides and proteins), surface-associated macromolecules, or quorum-sensing pathways rather than primarily driving membrane disruption. Thus, while more rigid amphipathic α-helices appear to favor direct antibacterial activity, a more flexible scaffold could support multifunctionality, particularly in the context of biofilm inhibition and host immune modulation. These findings highlight the importance of considering structural scaffold dynamics, in addition to physicochemical properties, in the rational design of multifunctional AMPs.

The cytotoxic effects of the peptides were evaluated on murine macrophages (RAW 264.7 cells). Cell viability was determined using the MTT (3-(4,5-dimethylthiazolyl-2)-2,5-diphenyl tetrazolium bromide) assay. None of the peptides had significant cytotoxicity up to 128 μmol L^–1^ (Figures 5A–C), indicating a favorable safety profile. Their potential immunomodulatory effects were also examined by measuring NO production in LPS-stimulated macrophages. At 16 μmol L^–1^, PcDBS1R1, PcDBS1R5, and PcDBS1R9 reduced NO production by 59%, 75%, and 78%, respectively (Figures 5D–F). However, PcDBS1R1 exhibited its strongest effect at 32 μmol L^–1^, achieving 73% NO reduction (Figure 5D). These results suggest that, in addition to their antibiofilm properties, these peptides may exert anti-inflammatory effects by modulating macrophage activation and NO production and, therefore, reducing cellular oxidative stress. Studies have shown that the marked upregulation of glycolysis and increased flux through the tricarboxylic acid (TCA) cycle in activated macrophages support the ATP citrate synthase–dependent generation of acetyl-coenzyme A. This metabolite drives histone acetylation and promotes the inducible expression of inflammatory genes.^33^ Subsequently, mitochondrial metabolism is disrupted through NO–mediated inhibition of key TCA cycle enzymes, such as aconitase 2. This metabolic impairment results in the accumulation of intermediates, including succinate and fumarate, which further sustain and amplify inflammatory responses.^34^ Interestingly, when it comes to NO modulation by AMPs, two main mechanisms can be cited, including endotoxin (e.g., LPS) sequestering and modulation of intracellular inflammatory pathways. In a previous work by our group, three peptides derived from a *Pyrobaculum aerophilum* ribosomal protein were studied in terms of structure, multiple biological activities, and mechanisms of action. In that work,^23^ the PaDBS1R7 peptide, as observed in the present work for PcDBS1R1, R5, and R9, displayed strong NO reduction in macrophages, also modulating pro-inflammatory cytokines. Additionally, to better understand how PaDBS1R7 led to this cellular oxidative stress reduction, CD experiments with LPS micelles were conducted at different peptide-to-LPS ratios. As a result, increasing LPS concentrations induced the formation of α-helix signatures, which were further supported by molecular docking and molecular dynamics simulations, thus confirming the potential of this peptide in sequestering free LPS and leading to NO expression reduction in murine macrophages. Moreover, in a recent work,^35^ researchers investigated the potential of self-assembled peptide-based fibrous hydrogel as a biological catalytic scaffold. In that work,^35^ the nanofibrous hydrogels could capture NO from external sources, retaining it within the interstitial spaces of their entangled fiber networks. At the same time, they exhibit anti-inflammatory activity in activated murine macrophages. This engineered peptide-based hydrogel system, designed for NO retention, offers valuable insights into the development of peptide biomaterials for biomedical applications.^35^

**Figure 5:**
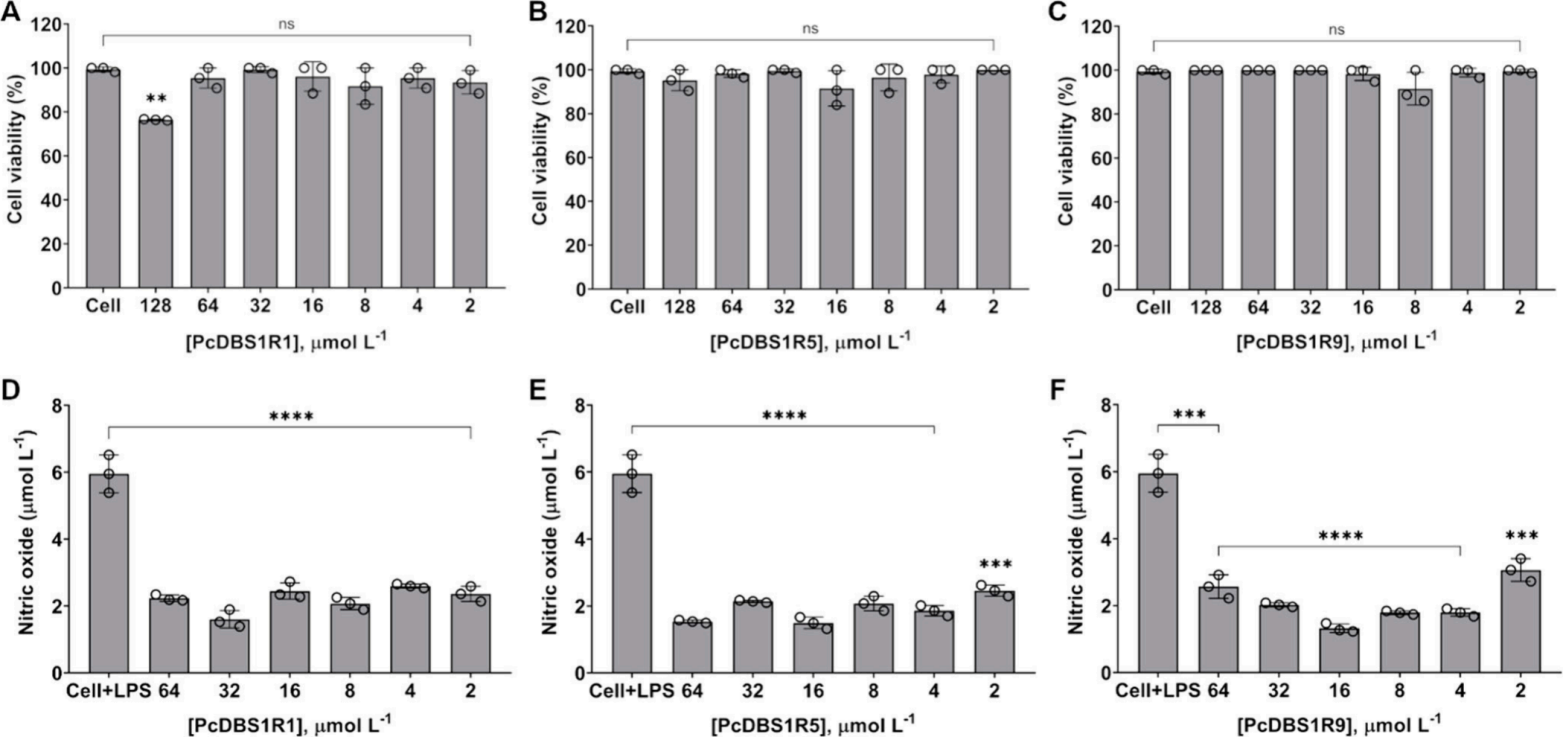
Cytotoxicity and nitric oxide modulation for the PcDBS1 derived peptides. Cell viability percentages of macrophages exposed at concentrations ranging from 128 to 2 μmol L^–1^ of PcDBS1R1 (A), PcDBS1R5 (B), and PcDBS1R9 (C). LPS-induced macrophages NO production in the presence of PcDBS1R1 (D), PcDBS1R5 (E), and PcDBS1R9 (F). Statistical significance was assessed using one-way analysis of variance (ANOVA), followed by Bonferroni’s multiple comparison tests (**p = 0.002, ***p = 0.0002, ****p < 0.0001). Error bars represent standard deviations. ns: not significant.

Importantly, all three peptides displayed negligible cytotoxicity toward murine macrophages, consistent with the safety profiles of other synthetic AMPs designed for therapeutic use. During the infection process, Gram-negative bacteria can secrete endotoxins (e.g., LPS) capable of triggering significant cellular stress and a subsequent increase in reactive oxygen species (ROS) production, including NO by immune cells, especially macrophages.^36^ Here, we found that the three PcDBS1 analogues tested cause significant reduction in NO production on LPS-stimulated macrophages, indicating a potential immunomodulatory or anti-inflammatory effect. Excessive NO release is associated with tissue damage and exacerbated inflammation.^37^ Therefore, the downregulation of NO production by these peptides could help mitigate host injury during infection. The dual functionality observed, antibiofilm and anti-inflammatory, suggests that these peptides may exert therapeutic effects beyond direct bacterial killing, contributing to host immune homeostasis.

It is worth noting that when it comes to host immune homeostasis, as described above, signals originating from diverse receptor classes converge on macrophage signaling pathways that initiate or enhance a broad spectrum of antibacterial responses. These include processes such as LC3-associated phagocytosis (LAP), metabolic reprogramming, and the generation of antimicrobial metabolites, as well as the formation of lipid droplets, activation of guanylate-binding proteins (GBPs), production of AMP, induction of metal ion–mediated toxicity, restriction of nutrient availability, autophagy, and NO synthesis. The latter (NO) is of particular interest as it is generated by inducible nitric oxide synthase (iNOS) to exert toxic effects on intracellular pathogens via free radical attack, causing lipid peroxidation and protein and DNA oxidation.^34,38^ Additionally, NO can react with reactive oxygen species (ROS) to form reactive nitrogen species (RNS), including highly reactive oxidants such as peroxynitrite, which exhibit strong antibacterial properties. These RNS are believed to target multiple classes of macromolecules. However, their ability to disrupt metalloproteins involved in essential processes such as DNA replication and cellular respiration is considered especially critical to their antimicrobial activity.^34^ Bearing this in mind, it is important to consider the well-balanced regulation of NO production by macrophages in the presence of AMPs to achieve the highest antimicrobial effect desired.

As a complement to the explanations and hypotheses outlined above, the concept of immunologically silent killing is closely associated with the finely tuned regulation of NO production, aimed at preventing excessive inflammatory responses during infection.^39^ Within this framework, we propose that our lead peptide candidates partially reduce NO production in RAW 264.7 macrophages (as shown in Figure 5D–F), while maintaining sufficient levels of this critical signaling molecule to support effective antibacterial responses. Nonetheless, further studies are required to evaluate the ability of these peptides to modulate pro-inflammatory cytokine production both *in vitro* and *in vivo* to fully characterize their immunomodulatory potential.

In summary, three *P. chabaudi*–derived peptides designed using the Joker algorithm exhibit distinct yet complementary structural and functional profiles. PcDBS1R5 and PcDBS1R9 have pronounced α-helical structures, strong amphipathicity, and robust antimicrobial and antibiofilm activities against *A. baumannii* clinical isolate. By contrast, PcDBS1R1, while less helical and more flexible, demonstrated selective antibiofilm activity and indications of immunomodulatory effects. The combination of antibiofilm (particularly against an *A. baumannii* clinical isolate), and NO modulatory effects, along with low cytotoxicity, supports the hypothesis that these peptides may function as multifunctional scaffolds with therapeutic potential. Nevertheless, it is important to highlight that future studies should aim to clarify the mechanisms of action governing membrane interactions, biofilm matrix targeting, and host immune modulation *in vitro* and *in vivo*.

## Supplementary Material



## Data Availability

All theoretical structures (atomic coordinates) generated for PcDBS1R1 – PcDBS1R9 using AlphaFold are available in the NOMAD repository (www.nomad-lab.eu) under the following NOMAD IDs: PcDBS1R1 (OcdtOrvJQ1KtEn_mmY2r5g), PcDBS1R2 (3uSjppHlQHWDIqz_acpGuw), PcDBS1R3 (5vmPBdBqRkyg4EF4wgfGvw), PcDBS1R4 (wpy6pKXuSPGa0obyoD0q2Q), PcDBS1R5 (tAPdvILuRQWo7nZeMcTKhg), PcDBS1R6 (LLFOfx6xTtm5gflcH1R5sQ), PcDBS1R7 (gCK0hOqtRXabYwwMCB0NYg), PcDBS1R8 (j2nLvEMgT3q69qBvcE7uWg), and PcDBS1R9 (R48HvREVQ46GtvBl0MA6cA).

## Notes

Structural validation scores, chemical shifts, mass spectrometry analysis, antibiogram, biophysical and biological assays and references (PDF)

## Notes

**Safety Statement.** No unexpected or unusually high safety hazards were encountered. All experiments involving clinical isolates of *A. baumannii* and *K. pneumoniae* were performed following appropriate biosafety practices and institutional guidelines. Circular dichroism and nuclear magnetic resonance were conducted using standard laboratory safety procedures. MTT cytotoxicity assays employing RAW cells were carried out in accordance with institutional regulations for animal-derived materials and cell culture handling.

## Abbreviations

AMPs: Antimicrobial peptides
DBS: Database sequence
GBPs: guanylate-binding proteins (GBPs)
iNOS: nitric oxide synthase
MIC: Minimal inhibitory concentration
MBC: Minimal bactericidal concentration
**LC**_**50**_: lethal concentration required to kill 50% of cells
CD: Circular dichroism
NMR: Nuclear magnetic resonance
ATCC: American Type Culture Collection
NO: Nitric oxide
MALDI-TOF MS: matrix-assisted laser desorption/ionization time-of-flight mass spectrometry
RNS: reactive nitrogen species
ROS: reactive oxygen species
SDS: sodium dodecyl sulfate
TFE: 2,2,2 trifluoroethanol
HRAN: Hospital regional asa norte
LACEN: Laboratório central de saúde pública
MTT: 3-(4,5-dimethylthiazolyl-2)-2,5-diphenyl tetrazolium bromide
LAP: LC3-associated phagocytosis
LPS: Lipopolysaccharide
TCA: tricarboxylic acid
