# Supplementary material for: Computer-Aided Design of Plasmodium chabaudi-Derived Peptides with Dual Antibiofilm and Anti-inflammatory Activities
Source: ACS Med Chem Lett. 2026 May 5;17(5):1057–66. doi: 10.1021/acsmedchemlett.6c00007 (PMC13181477; doi:10.1021/acsmedchemlett.6c00007)
Supplement: Supplementary file 1 [file ml6c00007_si_001.pdf]

SUPPORTING INFORMATION: Computer-aided design of *Plasmodium chabaudi* derived peptides with dual antibiofilm and anti-inflammatory activities

Raquel M. Quigua Orozco[1†], Alexandre D. O. Santos[1†], Joelma P. Rossetto[1†], Elisângela S. Madalozzo[2,3], Lívia V. Luchi[1], Samilla B. Rezende[1], Lai Yue Chan[4], Danieli F. Buccini[1], Maria L. R. Macedo[5], Angela Mehta[6], David J. Craik[4], Octávio L. Franco[1,7] and Marlon H. Cardoso[1,8*]

[1] S-Inova Biotech, Programa de Pós-Graduação em Biotecnologia, Universidade Católica Dom Bosco, Campo Grande, Brazil;

[2] Universidade Federal de Mato Grosso do Sul (UFMS), Campo Grande, Brazil;

[3] Universidade Estadual de Mato Grosso do Sul (UEMS), Naviraí, Brazil;

[4] Institute for Molecular Bioscience, Australian Research Council Centre of Excellence for Innovations in Peptide and Protein Science, The University of Queensland, Brisbane, Australia, 4072;

[5] Laboratório de Purificação de Proteínas e suas Funções Biológicas, Universidade Federal de Mato Grosso do Sul, Campo Grande, Brazil;

[6] Embrapa Recursos Genéticos e Biotecnologia, Brasília, Brazil;

[7] Centro de Análises Proteômicas e Bioquímicas, Programa de Pós-graduação em Ciências Genômicas e Biotecnologia, Universidade Católica de Brasília, Brasília, Brazil.

[8] Programa de Pós-Graduação em Ciências Ambientais e Sustentabilidade Agropecuária, Universidade Católica Dom Bosco, Campo Grande, Brazil.

[†]These authors contributed equally.

***Corresponding author:** Prof. Marlon H. Cardoso; Email: marlonhenrique6@gmail.com

Table of Contents

Supplementary Table 1: Structural validation scores for PcDBS1 peptide analogues predicted by AlphaFold2. The Z-score (ProSA-web), QMEAN4 score (SWISS-MODEL), and G-factor (PROCHECK) reflect energy profile, statistical quality, and stereochemical parameters, respectively…………………………………………………………………..3

Supplementary Table 2: Chemical shifts of PcDBS1R1 in 60% H_2O (v/v) 30% TFE-$_{d3}$ (v/v) and 10% D_2O (v/v)………………………………………………………………………………4

Supplementary Table 3: Chemical shifts of PcDBS1R5 in 60% H_2O (v/v) 30% TFE-$_{d3}$ (v/v) and 10% D_2O (v/v)………………………………………………………………………………5

Supplementary Table 4: Chemical shifts of PcDBS1R9 in 60% H_2O (v/v) 30% TFE-$_{d3}$ (v/v) and 10% D_2O (v/v)………………………………………………………………………………6

Supplementary Figure 1: Mass spectrometry analysis of PcDBS1R1. Ion of monoisotopic mass [M+H]+ of 2120.44 m/z is represented…………………………………………………7

Supplementary Figure 2: Mass spectrometry analysis of PcDBS1R5. Ion of monoisotopic mass [M+H]+ of 2135.17 m/z is represented…………………………………………………8

Supplementary Figure 3: Mass spectrometry analysis of PcDBS1R9. Ion of monoisotopic mass [M+H]+ of 2147.26 m/z is represented…………………………………………………9

Supplementary Figure 4: Antibiogram of *Acinetobacter baumannii* and *Klebsiella pneumoniae* clinical isolates against eleven antibiotics. Heatmaps showing the percentage of *A. baumannii* and *K. pneumoniae* clinical isolates growth inhibition in the presence of penicillin, amoxicillin, gentamicin, ampicillin, chloramphenicol, kanamycin, cefepime, imipenem, cefaclor, meropenem and ciprofloxacin at concentration ranging from 2 to 64 μmol L^{-1}…………………………………………………………………………………10

Biophysical, biological assays and references………………………………………11-17

Table S1. Structural validation scores for PcDBS1 peptide analogues predicted by AlphaFold2. The Z-score (ProSA-web), QMEAN4 score (SWISS-MODEL), and G-factor (PROCHECK) reflect energy profile, statistical quality, and stereochemical parameters, respectively.

	**Z-score[a]**	**QMEAN4[b]**	**G-factor**
**PcDBS1R1**	0.26	-0.83	0.05
**PcDBS1R2**	-1.06	0.62	0.21
**PcDBS1R3**	0.18	-1.26	-0.41
**PcDBS1R4**	-0.3	0.05	-0.01
**PcDBS1R5**	-1.36	-0.14	-0.18
**PcDBS1R6**	-0.97	0.78	-0.30
**PcDBS1R7**	-0.14	-0.99	-0.31
**PcDBS1R8**	-1.44	0.52	-0.22
**PcDBS1R9**	-1.24	0.03	0.04

[a]Z- score within the expected values for NMR structures deposited in the PDB with similar size and fold compared to generated structures[1].

[b]Values above −1.75 are considered acceptable[2].

Table S2. Chemical shifts of PcDBS1R1 in 60% H_2O (v/v) 30% TFE-$_{d3}$ (v/v) and 10% D_2O (v/v).

	NH	Hα	Hβ	Others
**Pro**	-	4.45	1.99, 2.49	αCH2 2.11, δCH2 3.45
**Lys**	8.71	4.37	1.79, 1.89	γCH2 1.44; δCH2 1.69, 1.72; εCH2 3.01, 3.03
**Leu**	8.25	4.32	1.61, 1.63	γCH 1.57; δCH3 0.93
**Ala**	8.19	4.31	1.40	
**Ile**	7.72	4.09	1.90, 1.21	γCH2 1.53; δCH3 0.90
**Arg**	8.07	4.30	1.83, 1.88	γCH2 1.59; δCH2 3.18, 3.20
**Ile**	8.12	4.09	1.89, 1.19	γCH2 1.52; δCH3 0.84
**Thr**	8.06	4.22	4.24, 1.23	
**Cys**	8.21	4.40	2.97, 3.01	
**Lys**	8.12	4.25	1.86, 1.92	γCH2 1.42; δCH2 1.68, 1.72; εCH2 2.98, 2.99
**Ile**	7.98	4.03	1.88, 1.19	γCH2 1.48; δCH3 0.81
**His**	8.26	4.60	3.23, 3.32	
**Lys**	8.07	4.27	1.84, 1.86	γCH2 1.50, 1.69; δCH2 1.72; εCH2 2.99, 3.0
**Lys**	8.24	4.34	1.81, 1.87	γCH2 1.51; δCH2 1.70, 1.72
**Val**	8.05	4.17	2.09, 0.93	γCH3 1.52
**Ala**	8.17	4.44	1.39	
**Ile**	7.89	4.31	1.89, 1.19	γCH2 1.49; δCH3 0.87
**Ser**	8.14	4.59	3.85, 3.88	
**Val**	7.79	4.23	2.17, 0.92	γCH3 0.94

Table S3. Chemical shifts of PcDBS1R5 in 60% H_2O (v/v) 30% TFE-$_{d3}$ (v/v) and 10% D_2O (v/v).

	NH	Hα	Hβ	Others
**Pro**		4.46	2.07, 2.54	αCH_2 2.11, 2.15; δCH_2 3.45, 3.47
**Met**	8.83	4.53	2.08, 2.11	γCH_2 2.60, 2,62; εCH_3 2.12
**Asn**	8.29	4.66	2.86, 2.93	
**Ala**	8.30	4.16	1.48	
**Ile**	7.67	3.90	2.02, 1.34	γCH_2 0.96; δCH_3 0,89
**Lys**	7.81	4.02	1.76, 1.89	γCH_2 1.43, 1.47; δCH_2 1.61, 1.63; εCH_2 2.98, 3.0
**Leu**	7.67	4.18	1.82, 1.85	γCH 1.73; δCH_3 0.91, 0.97
**Leu**	8.27	4.10	1.81, 1.94	γCH 1.62; δCH_3 0.91, 1.04
**Cys**	8.30	4.26	3.11, 3.16	
**Arg**	7.88	4.20	1.86, 2.07	γCH_2 1.71, 1.74
**Val**	8.25	3.86	2.19, 0.91	γCH_3 1.04
**His**	8.32	4.47	3.30, 3.39	
**Lys**	7.97	4.21	1.92, 1.97	γCH_2 1.51, 1.52; δCH_2 1.73, 1,76; εCH_2 3.00, 3.02
**Lys**	8.08	4.26	1.88, 1.99	γCH_2 1.49, 1.51; δCH_2 1.69, 1.74; εCH_2 3.00, 3.02
**Ile**	8.02	4.08	1.90, 1.19	γCH_2 1.58; γCH_3 0.93; δCH_3 0.87
**Ala**	8.06	4.33	1.41	
**Ile**	7.79	4.31	1.98, 1.29	γCH_2 1.52, γCH_3 0.97; δCH_3 0.87
**Ser**	8.04	4.58	3.90, 3.93	
**Val**	7.73	4.23	2.20, 0.95	γCH_3 0.98

Table S4. Chemical shifts of PcDBS1R9 in 60% H_2O (v/v) 30% TFE-$_{d3}$ (v/v) and 10% D_2O (v/v).

	NH	Hα	Hβ	Others
**Pro**		4.46	2.07, 2.54	αCH_2 2.11, 2.15; δCH_2 3.45, 3.47
**Met**	8.83	4.53	2.08, 2.11	γCH_2 2.60, 2.62; εCH_3 2.12
**Asn**	8.29	4.66	2.86, 2.93	
**Ala**	8.30	4.16	1.48	
**Ile**	7.67	3.90	2.02, 1.34	γCH_3 0.96; δCH_3 0.89
**Lys**	7.81	4.02	1.76, 1.89	γCH_2 1.43, 1.47; δCH_2 1.61, 1.63; εCH_2 2.98, 3.0
**Leu**	7.67	4.18	1.82, 1.85	γCH 1.73; δCH_3 0.91. 0.97
**Leu**	8.27	4.10	1.81, 1.94	γCH 1.62; δCH_3 0.91, 1.04
**Cys**	8.30	4.26	3.11, 3.16	
**Arg**	7.88	4.20	1.86, 2.07	γCH_2 1.71, 1.74
**Val**	8.25	3.86	2.19, 0.91	γCH_3 1.04
**His**	8.32	4.47	3.30, 3.39	
**Lys**	7.97	4.21	1.92, 1.97	γCH_2 1.51, 1.52; δCH_2 1.73, 1.76; εCH_2 3.00, 3.02
**Lys**	8.08	4.26	1.88, 1.99	γCH_2 1.49, 1.51; δCH_2 1.69, 1.74; εCH_2 3.00, 3.02
**Ile**	8.02	4.08	1.90, 1.19	γCH_3 1.58; γCH_2 0.93; δCH_3 0.87
**Ala**	8.06	4.33	1.41	
**Ile**	7.79	4.31	1.98, 1.29	γCH_3 1.52; γCH_2 0.97; δCH_3 0.87
**Ser**	8.04	4.58	3.90, 3.93	
**Val**	7.73	4.23	2.20, 0.95	γCH_3 0.98

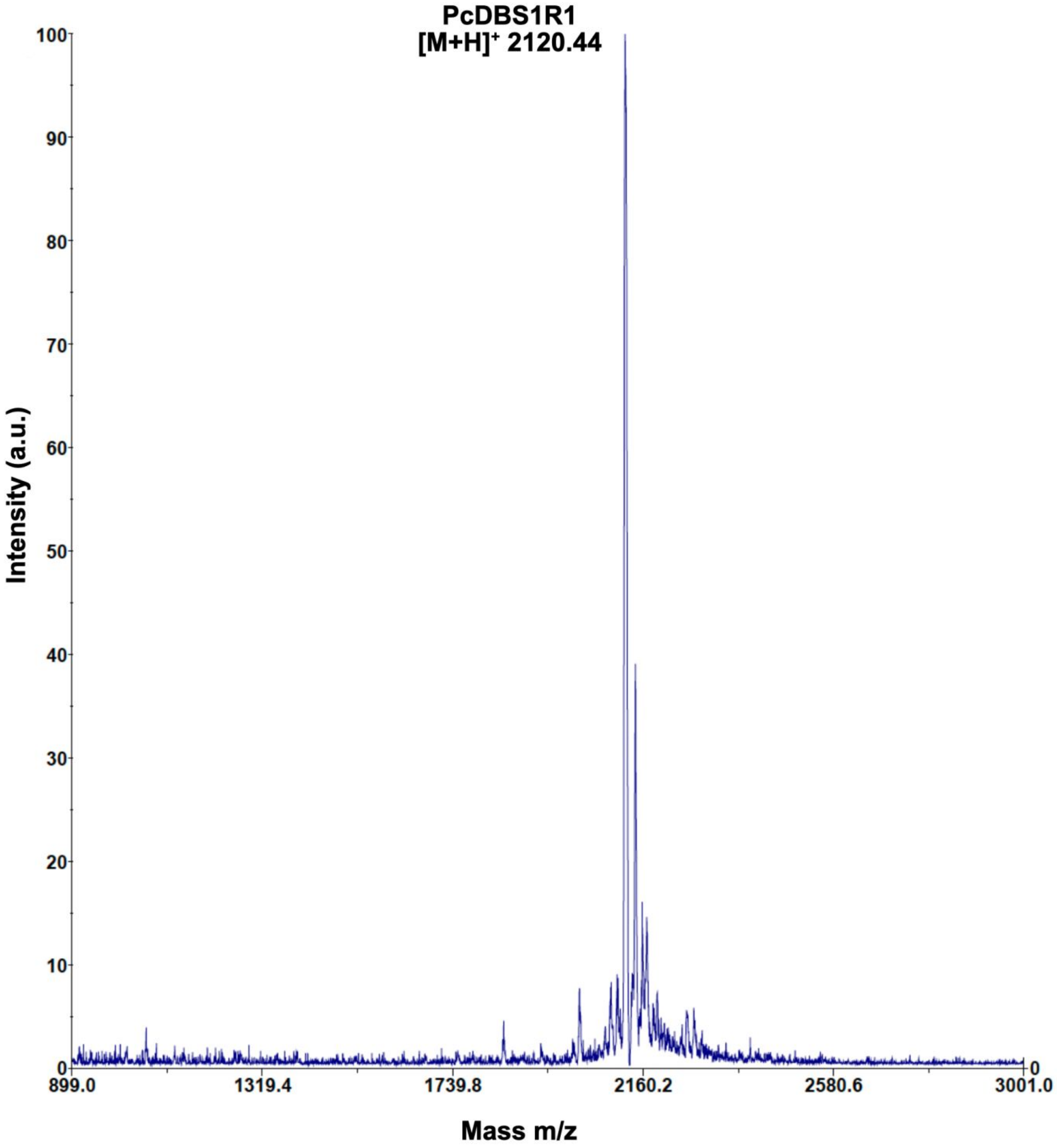

Supplementary Figure 1: Mass spectrometry analysis of PcDBS1R1. Ion of monoisotopic mass $[M+H]^+$ of 2120.44 m/z is represented.

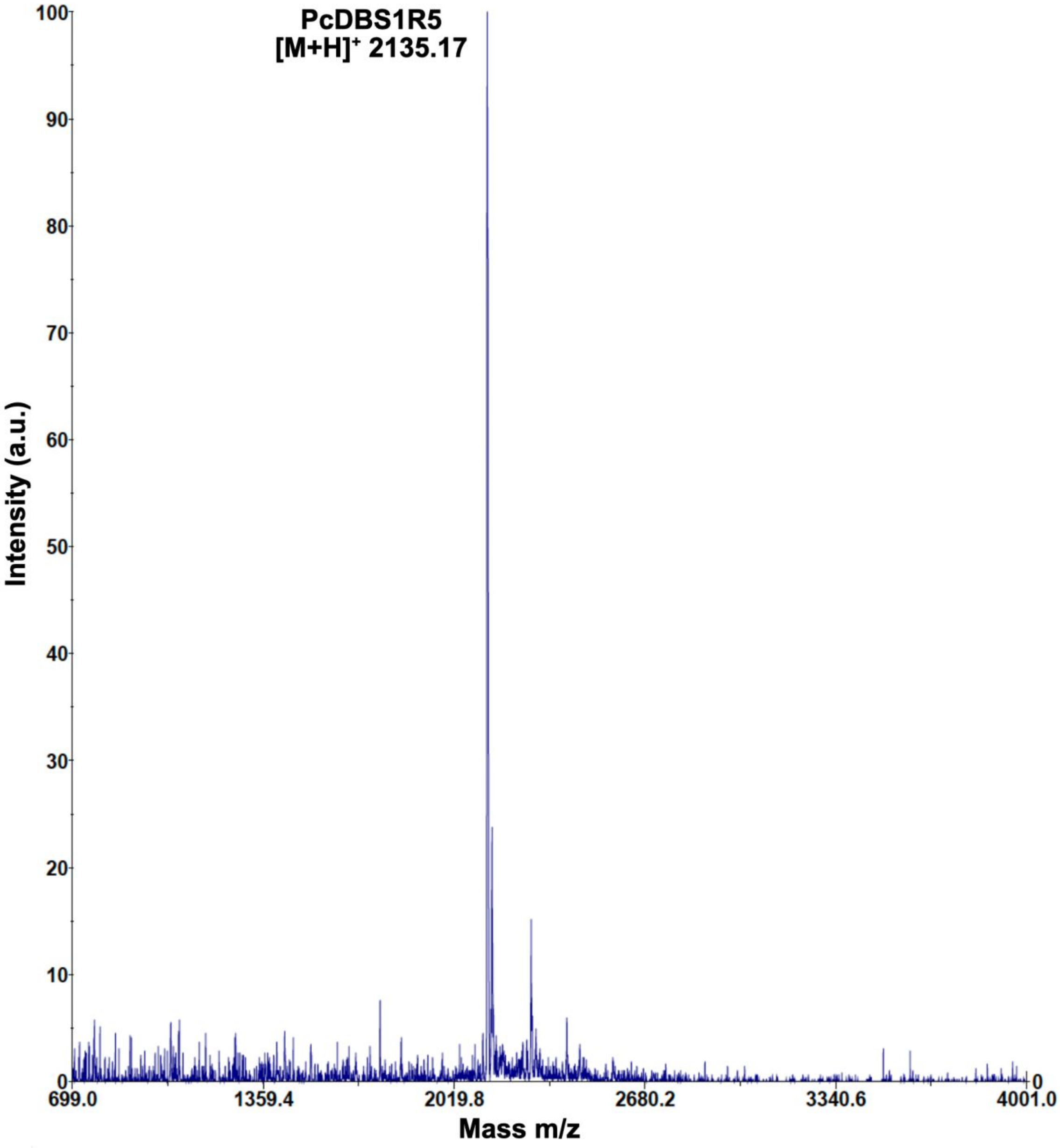

Supplementary Figure 2: Mass spectrometry analysis of PcDBS1R5. Ion of monoisotopic mass $[M+H]^+$ of 2135.17 m/z is represented.

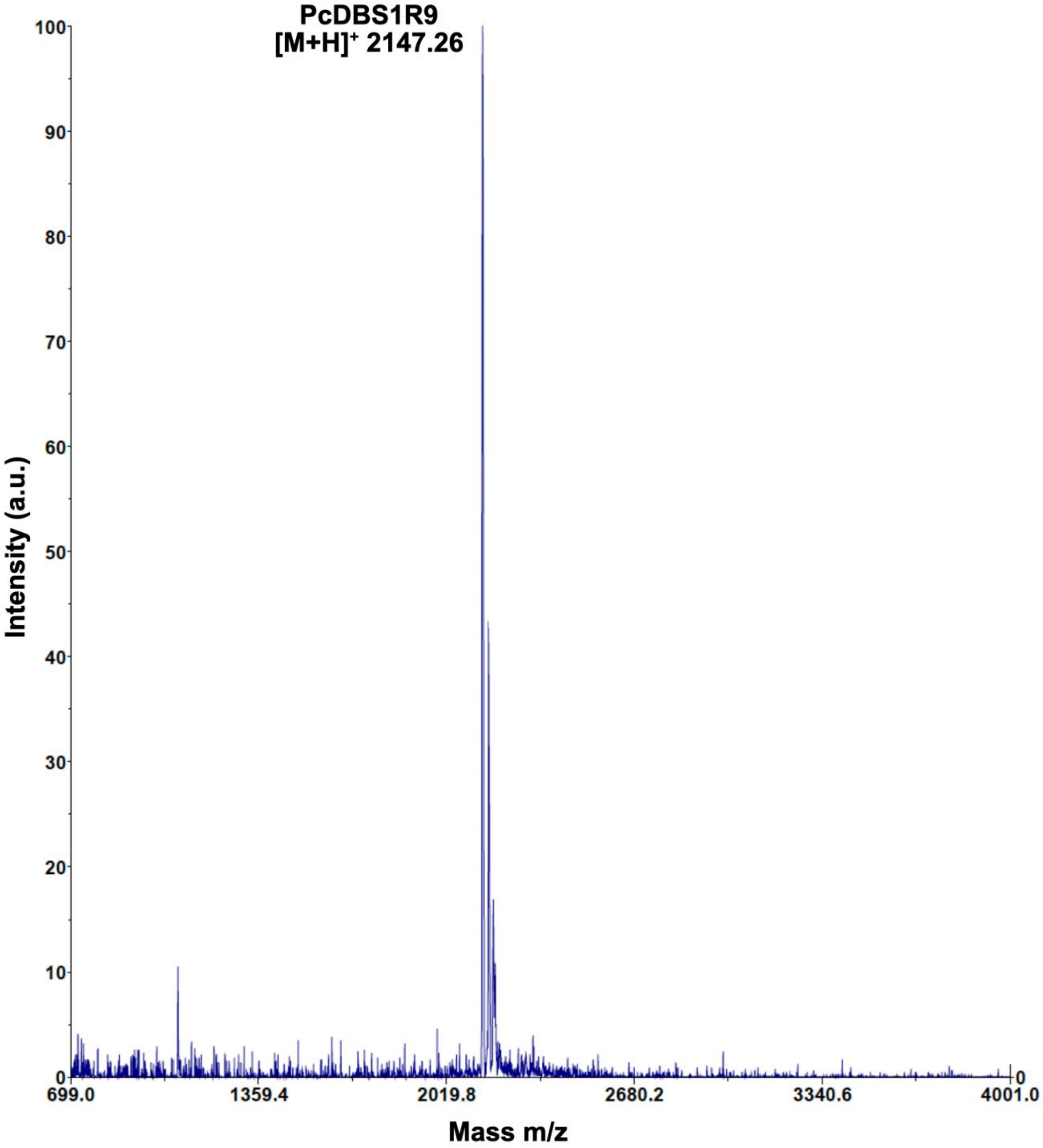

Supplementary Figure 3: Mass spectrometry analysis of PcDBS1R9. Ion of monoisotopic mass $[M+H]^+$ of 2147.26 m/z is represented.

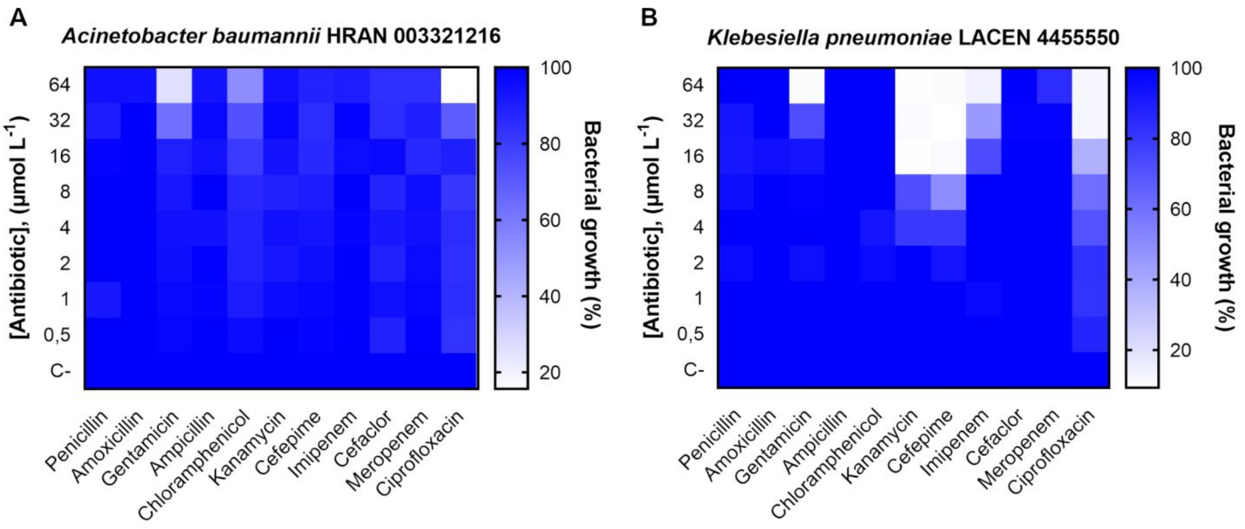

Supplementary Figure 4: Antibiogram of *Acinetobacter baumannii* and *Klebsiella pneumoniae* clinical isolates against eleven antibiotics. Heatmaps showing the growth percentages of *A. baumannii* and *K. pneumoniae* clinical isolates in the presence of penicillin, amoxicillin, gentamicin, ampicillin, chloramphenicol, kanamycin, cefepime, imipenem, cefaclor, meropenem, and ciprofloxacin at concentrations ranging from 2 to 64 μmol L^{-1}.

Materials and methods

Molecular modeling

Three-dimensional theoretical models for the PcDBS1 derivatives were generated by submitting the corresponding peptide sequences to the AlphaFold2 server[3]. For each peptide, the software generated and ranked the five best structural models according to their free-energy scores. The models with the lowest free-energy value were subsequently validated regarding their stereochemical parameters and fold quality using PROCHECK[4], ProSA-web[1], and QMEAN4[2]. Structure visualization was done in PyMOL 2.3[5].

Peptides synthesis

The PcDBSR1, R5 and R9 peptides were synthesized with >95% purity using the N-9-fluorenylmethyloxycarbonyl (Fmoc) technology. The molecular masses were verified by mass spectrometry using a MALDI-TOF/MS calibrated using Peptide Calibration Standard II (Bruker Daltonics) as molecular mass standards. The peptides were purchased from Peptide 2.0 Incorporated (Chantilly, VA, USA).

Circular dichroism spectroscopy

CD spectroscopy was performed in ultrapure water, 10 mmol L^{-1} potassium phosphate buffer (pH 7.2), 50 mmol L^{-1} sodium dodecyl sulfate (SDS) micelles, and 2,2,2-trifluoroethanol (TFE) 30% in water (v/v). CD measurements were carried out on a JASCO spectropolarimeter J-1100 (Tokyo, Japan) equipped with a Peltier temperature controller (25°C), using cuvettes of 1 mm path length. CD spectra were recorded from 185 to 260 nm, at 25°C, with data pitch of 0.5 nm, at a scan speed of 50 nm min^{-1}. The resolution was 0.1 nm with a 1 s response time and five scan accumulations for each sample. The measurements were conducted at a peptide concentration of 50 µmol L^{-1}. The CD signals obtained for the solvents alone were subtracted from all spectra containing peptide

solutions. All spectra were smoothed using the Jasco Fast Fourier transform algorithm and baseline-corrected. The helix content of each peptide was calculated from the ellipticity values at 222 nm, as described by Chen and coworkers[6].

Nuclear magnetic resonance

All NMR spectra were acquired at a final peptide concentration of 1 mmol L^{-1} in a solvent mixture of 60% H_2O, 30% TFE-d_3, and 10% D_2O (v/v) at pH 4.3, as described by Cardoso and coworkers[7]. 4,4-Dimethyl-4-silapentane-1-sulfonic acid (DSS) was used as the internal chemical shift reference. One-dimensional ^1H spectra and two-dimensional spectra, including TOCSY and NOESY, were recorded at 298 K on a Bruker Avance 600 MHz spectrometer. Spectral data were processed with TopSpin 2.1 (Bruker) and resonance assignment was performed using CCPNMR Analysis 2.4[8]. Amino acid spin systems were assigned according to established procedures[9]. Secondary chemical shifts for Hα were obtained by subtracting the random-coil values from the experimental ^1H NMR chemical shifts[9]. Temperature coefficient experiments were performed in 70% (v/v) D_2O and 30% (v/v) TFE-d_3 at pH 4.3. Spectra were recorded over a temperature range of 285–310 K, with 5 K increments. Amide proton temperature coefficients more positive than −4.6 ppb K^{-1} were considered indicative of intrapeptide hydrogen bonding.

Bacterial strains

A. baumannii ATCC 19906 and *K. pneumoniae* ATCC 10031 were acquired from the American Type Culture Collection (ATCC). Additionally, a clinical isolate of *A. baumannii* (003321216) was provided by the Hospital Regional Asa Norte de Brasília (HRAN), and a clinical isolate of *K. pneumoniae* (4455550) was obtained from the Laboratório Central de Brasília (LACEN-DF). All bacterial strains were preserved in 20% (v/v) glycerol at −20 °C until use. Antibiogram assays were performed with *A. baumannii* (003321216) and *K.*

pneumoniae (4455550) clinical isolates against penicillin, amoxicillin, gentamicin, ampicillin, chloramphenicol, kanamycin, cefepime, imipenem, cefaclor, meropenem and ciprofloxacin at concentration ranging from 64 to 2 μmol L^{-1}, following the 96-well microplate dilution protocol established by the Clinical and Laboratory Standards Institute[10].

Bacteriostatic and bactericidal effects of the peptides against planktonic bacteria

Antimicrobial assays were performed against *A. baumannii* and *K. pneumoniae* strains, following the 96-well microplate dilution protocol established by the Clinical and Laboratory Standards Institute[10]. Bacteria were initially cultured on Mueller-Hinton agar (MHA) and incubated at 37 °C for 18 h. Three isolated colonies of each strain were inoculated into 5 mL of Mueller Hinton broth (MHB) and incubated at 37 °C, at 200 rpm, overnight. Then, the cultures were diluted 1:50 in fresh MHB and incubated at 37 °C until reaching logarithmic growth, monitored spectrophotometrically at 600 nm. The final bacterial concentration was adjusted to 5×10^5 CFU mL^{-1}, and peptides were tested at concentrations ranging from 64 to 2 µmol L^{-1}. Ciprofloxacin, used at the same concentrations, served as the positive control, whereas untreated bacterial suspensions (5×10^5 CFU mL^{-1} in MHB) were used as the negative control. The 96-well microplates were incubated at 37 °C for 18 h, and optical density (O.D.) readings were taken at 600 nm. The results were expressed as the percentages of bacterial planktonic cell growth inhibition in the presence of the peptides. Bactericidal effects were determined by plating 10 µL from wells without visible growth onto MHA plates, followed by incubation at 37 °C, for 24 h. The bactericidal activity was defined as the lowest peptide concentration resulting in the complete absence of bacterial colony formation. All tests were performed with three biological replicates.

Biofilm quantification assays

To identify the most suitable broth medium for biofilm formation, quantification assays were performed using clinical isolates of *A. baumannii* (HRAN 003321216) and *K. pneumoniae* (LACEN 4455550). Bacterial strains were initially plated on MHA plates and incubated at 37 °C, for 18 h. Three isolated colonies from each strain were then inoculated into 5 mL of MHB and incubated at 37 °C and 200 rpm for 18 h. Then, the resulting cultures were diluted 1:100 in three different media, Basal Medium 2 (BM2), Tryptic Soy Broth (TSB), and Brain Heart Infusion (BHI), and transferred to round-bottom 96-well microplates, followed by incubation at 37 °C for 24 h. Wells containing only broth served as negative controls. After incubation, planktonic cells were removed by washing the wells with distilled water. Biofilms were stained with 0.1% crystal violet for 20 min, followed by washing to remove excess dye. The adherent stained cells were resuspended in 60% ethanol, and absorbance was measured at 595 nm using a microplate reader to quantify biofilm biomass.

Biofilm inhibition assays

Biofilm inhibition was assessed using clinical isolates of *A. baumannii* (HRAN 003321216) and *K. pneumoniae* (LACEN 4455550). Bacterial strains were cultured on MHA plates and incubated at 37 °C for 18 h. Three isolated colonies of each strain were subsequently inoculated into 5 mL of MHB and incubated overnight at 37 °C and 200 rpm. The resulting cultures were diluted 1:50 in BM2 and transferred to round-bottom 96-well microplates at a final inoculum ratio of 1:100 (bacteria/broth). Plates were incubated at 37 °C for 24 h in the presence of peptides at concentrations ranging from 64 to 2 μmol L^{-1}. Ciprofloxacin, tested at the same concentrations, was used as the positive control, whereas untreated bacterial suspensions in BM2 served as the biofilm formation control. The experiments were performed with three biological replicates. After incubation, biofilm formation was evaluated using the crystal violet staining method, as described above. Adherent cells were stained

with 0.1% crystal violet, and absorbance was measured at 595 nm using a microplate reader.

Cell viability assay

Murine macrophages (RAW 264.7) were cultivated in Dulbecco's modified Eagle's medium (DMEM, Vitrocell) supplemented with 10% fetal bovine serum (FBS, Sigma-Aldrich) and 1% (v/v) penicillin–streptomycin (5000 U mL^{-1}; Life Technologies). To evaluate the cytotoxic effects of peptides PcDBS1R1, R5, and R9, 1 × 10^5 cells per well (200 μL) were seeded into 96-well plate and incubated at 37°C, 5% CO_2 atmosphere for 24 h to allow adhesion. Cells were then exposed to peptide concentrations ranging from 128 to 2 μmol L^{-1} per well and incubated for 24 h under the same conditions. Cell viability was determined using the MTT (3-(4,5-dimethylthiazolyl-2)-2,5-diphenyl tetrazolium bromide) assay. Briefly, MTT solution (5 mg mL^{-1} in PBS) was added to each well, followed by incubation for 4 h. After incubation, the MTT-peptide solution was removed, and a 60 μL alcohol/chloride acid solution was added. Untreated cells served as the control. All assays were done in triplicates. Absorbance was measured at 600 nm using a microplate reader (BioTek PowerWave XS), and cell viability was calculated according to the following equation:

$$\%\ \text{Cell viability} = \frac{\text{Optical density of treated cells}}{\text{Optical density of untreated cells}}\ \text{x}\ 100$$

Nitric oxide production quantification

NO production was quantified following the Griess method[11], with modifications. RAW 264.7 macrophages (2 × 10^5 cell mL^{-1}) were incubated in DMEM for 24 h in the presence of LPS at 1 mg mL^{-1}, with or without peptides at concentrations ranging from 64 to 2 μmol L^{-1}. Cells with LPS alone were used as positive control, whereas untreated cells served as negative

control. After incubation, 50 µL of the supernatants were collected and quantified with Griess reagent. Absorbance was measured at 570 nm using a microplate reader (BioTek PowerWave XS). Nitrite concentrations were determined based on a standard calibration curve (200 – 1 µmol L^{-1}). The assays were carried out in triplicate.

Statistical analysis

Statistical analyses were performed using one-way ANOVA followed by Bonferroni's multiple comparison test to determine the difference between inhibition of the different concentrations of the peptides used. Data are presented as mean ± standard deviation (SD). Statistical significance was defined as $p < 0.05$. All analyses were conducted using GraphPad Prism (version 8.0.1; GraphPad Software, Inc., San Diego, CA, USA).

References

(1) Wiederstein, M.; Sippl, M. J. ProSA-Web: Interactive Web Service for the Recognition of Errors in Three-Dimensional Structures of Proteins. *Nucleic Acids Res* 2007, *35* (SUPPL.2). https://doi.org/10.1093/nar/gkm290.

(2) Benkert, P.; Biasini, M.; Schwede, T. Toward the Estimation of the Absolute Quality of Individual Protein Structure Models. *Bioinformatics* 2011, *27* (3), 343–350. https://doi.org/10.1093/bioinformatics/btq662.

(3) Mirdita, M.; Schütze, K.; Moriwaki, Y.; Heo, L.; Ovchinnikov, S.; Steinegger, M. ColabFold: Making Protein Folding Accessible to All. *Nat Methods* 2022, *19* (6), 679–682. https://doi.org/10.1038/s41592-022-01488-1.

(4) Laskowski, R. A.; MacArthur, M. W.; Moss, D. S.; Thornton, J. M. PROCHECK: A Program to Check the Stereochemical Quality of Protein Structures. *J. Appl. Cryst.* 1993, *26*, 283–291. https://doi.org/10.1107/S0021889892009944.

(5) Schrödinger LLC. *The PyMOL Molecular Graphics System, Version~1.8*; 2015.

(6) Chen, Y.-H.; Yang, J. T.; Chau, K. H. Determination of the Helix and β Form of Proteins in Aqueous Solution by Circular Dichroism. *Biochemistry* 1974, *13* (16), 3350–3359. https://doi.org/10.1021/bi00713a027.

(7) Cardoso, M. H.; Cândido, E. S.; Chan, L. Y.; Torres, M. D. T.; Oshiro, K. G. N.; Rezende, S. B.; Porto, W. F.; Lu, T. K.; De La Fuente-Nunez, C.; Craik, D. J.; Franco, O. L. A Computationally Designed Peptide Derived from Escherichia Coli as a

Potential Drug Template for Antibacterial and Antibiofilm Therapies. *ACS Infect Dis* 2018, *4* (12), 1727–1736. https://doi.org/10.1021/acsinfecdis.8b00219.

(8) Vranken, W. F.; Boucher, W.; Stevens, T. J.; Fogh, R. H.; Pajon, A.; Llinas, M.; Ulrich, E. L.; Markley, J. L.; Ionides, J.; Laue, E. D. The CCPN Data Model for NMR Spectroscopy: Development of a Software Pipeline. *Proteins: Structure, Function, and Bioinformatics* 2005, *59* (4), 687–696. https://doi.org/https://doi.org/10.1002/prot.20449.

(9) Wishart, D. S.; Bigam, C. G.; Holm, A.; Hodges, R. S.; Sykes, B. D. 1H, 13C and 15N Random Coil NMR Chemical Shifts of the Common Amino Acids. I. Investigations of Nearest-Neighbor Effects. *J Biomol NMR* 1995, *5* (1), 67–81. https://doi.org/10.1007/BF00227471.

(10) Clinical and Laboratory Standards Institute. *Performance Standards for Antimicrobial Susceptibility Testing. 30th Ed. CLSI Suplement M100*; 2020; Vol. 40.

(11) Green, L. C.; Wagner, D. A.; Glogowski, J.; Skipper, P. L.; Wishnok, J. S.; Tannenbaum, S. R. Analysis of Nitrate, Nitrite, and [15N]Nitrate in Biological Fluids. *Anal Biochem* 1982, *126* (1), 131–138. https://doi.org/https://doi.org/10.1016/0003-2697(82)90118-X.
